# Prognostic Markers of Diabetic Foot Ulcer Healing in A Resource‐Limited Setting

**DOI:** 10.1111/iwj.71009

**Published:** 2026-08-07

**Authors:** Roozbeh Naemi, Zulfiqarali G. Abbas

**Affiliations:** ^1^ School of Health and Society University of Salford Manchester UK; ^2^ Abbas Medical Centre Dar es Salaam Tanzania; ^3^ Muhimbili University of Health and Allied Sciences Dar es Salaam Tanzania; ^4^ University of Staffordshire Stoke‐on‐Trent UK

**Keywords:** Africa, diabetes neuropathies, diabetic foot, foot ulcer, wound healing

## Abstract

This study aimed to identify the prognostic markers for Diabetic Foot Ulcer (DFU) healing using routinely collected clinical data. A prospective cohort study of 1075 (M/F: 664/411) patients who attended the foot clinic with a DFU was conducted. At baseline, detailed clinical data were collected and ulcer characteristics were systematically assessed and classified. During 167 ± 408 days follow‐up, ulcers of 1039 patients healed and 36 did not. Ulcers with SINBAD classifications 4, 5 and 6 (compared to SINBAD‐3) showed 50%, 60% and 70% increase in the risk of not healing and 2.2, 2.8 and 3.3 folds increase in healing duration respectively. Also, deeper ulcer and having previous ulcer history showed 68% and 47% increase in the risk of ulcer not healing and 57% and 20% increase in healing duration respectively. Each day delay in presentation increased the risk of ulcer not healing by 0.5%. The duration of healing was 2.4 times longer for bigger ulcers (> 1 cm^2^) and 24% longer in patients with peripheral arterial disease. SINBAD score was the strongest predictor of ulcer healing. Among SINBAD constitutive components, ulcer depth (deep‐vs‐shallow) was the strongest predictor of risk of ulcer not healing, while ulcer size (bigger‐vs‐smaller than 1 cm^2^) was the strongest predictor of ulcer healing duration.

## Key Points

- The SINBAD score is the strongest prognostic marker of diabetic foot ulcer healing, with ulcer depth being the strongest predictor of non‐healing and ulcer size the strongest predictor of time to heal.

## 1 Introduction

Diabetic Foot Ulcers lifetime incidence was estimated to be between 15% and 25% [1] and was reported to be between 19% and 34% in people with diabetes at any point in time [2]. The rate of ulcer recurrence was also approximated to be 65% in 5 years [2], with more than 50% of diabetic ulcers becoming infected [3] with approximately 1 in 5 moderate or severe diabetic foot infections leading to amputation [4, 5]. In fact, globally, diabetic Foot Ulcer (DFU) is the main cause of non‐traumatic lower limb amputation worldwide [1] and it was established that the DFU increases the risk of death at 5 years by 2.5 times [6]. To decrease the socioeconomic burden of DFU, an understanding of the prognostic markers in the prediction of DFU wound healing is necessary.

A previous study (EURODIALE) has identified older age, male sex, heart failure, the inability to stand or walk without help, end‐stage renal disease, larger ulcer size, peripheral neuropathy (PN) and Peripheral Arterial Disease (PAD) as independent baseline predictors of non‐healing ulcers [7].

Earlier studies identified wound size, wound duration and ulcer grade as predictors of healing [8]. More recent work using a larger DFU cohort has shown that healing outcomes are influenced by wound age, wound size, the number of concurrent wounds, infection, patient age, Wagner grade, ambulatory status, renal dialysis or transplant history, peripheral arterial disease and any cause of hospitalisation [9].

The IWGF guidelines promote classification and scoring systems to help both clinical management and audit the outcomes of routine care in patients with diabetic foot ulcer [10, 11, 12].

While the IWGDF wound classification guideline recommends using simple, validated systems—primarily SINBAD due to its practicality and consistency across settings, it concludes that no current system can reliably predict outcomes for an individual ulcer [13].

A recent multi‐clinic study identified presentation delay, prior minor amputation, ulcer location, surface area and ischaemia as independent predictors of DFU healing [14]. This five‐variable model outperformed models based on existing classification systems [14].

Despite these, there is a dearth of studies in which the factors contributing to diabetic foot ulcer healing are studied in resource‐constraint settings where Diabetes imposes a heavy burden on the health services [15]. The prevalence of diabetic foot complications in Tanzanian populations has been previously reported in detail and a high mortality rate of 29% was reported among patients with foot ulcer [16]. The estimated expenses to both the patient and society for treating DFU differ from other settings [16, 17, 18, 19].

In previously conducted cohort studies we reported on the risk factors of diabetic foot ulceration [20] and on common characteristics of patients with and without active diabetic foot ulcer [21] in low resource settings. However, to reduce the socioeconomic burden of diabetic foot complications, it is essential to understand the prognostic factors that determine the DFU healing efficacy. In this context, it is important to identify the factors contributing to DFU healing efficacy, not only those associated with non‐healing, but also those that determine healing time, which carries significant socio‐economic implications, particularly in LMIC settings.

The overall aim of this study was to identify the prognostic factors associated with the diabetic foot ulcer healing efficiency.

The first objective was to identify the variables that determine the healing outcome as to whether those can predict if a DFU heals or remains unhealed. The second objective was to identify the variables that affect the DFU healing time.

## 2 Materials and Methods

### 2.1 Participants

In this prospective cohort study data from 1174 patients with an active foot ulcer who attended the specialist diabetes service in a single diabetes clinic in Dar es Salaam, Tanzania between January 2014 to December 2021 was utilised. The primary inclusion criteria were that the patient should be diagnosed with diabetes and the presence of DFU at baseline. DFU was defined as a full‐thickness wound involving the foot or the ankle, distal to or including the malleoli. The primary exclusion criteria were the presence of major lower limb amputation. All the patients on registration were taken full history, demographic details and full examination for PAD, PN and wound.

Analysis was conducted by following participants very closely to establish healing efficacy including whether the DFU healed or not (censored) and the duration of treatment (healed or not healed—censored).

### 2.2 Data Collection

A set of 15 measures was collected from the participants during a single visit at baseline. These are described as follows:

#### 2.2.1 Patient‐Related Measures

The patient related categorical measures were: Gender, smoking (Current smoker, Never smoked, Previous smoker), previous amputation and history of ulceration, according to protocols set by IWGDF [22]. Peripheral artery disease (PAD) was defined according to IWGFD guidelines as obstructive atherosclerotic vascular disease of the arteries from aorta to foot with clinical symptoms, signs or abnormalities, resulting in disturbed or impaired circulation in one or more extremities.

The presence of PAD was also diagnosed based on the protocol proposed in IWGFD guidelines for a person with diabetes and a foot ulcer which recommends to take a relevant history for peripheral artery disease, examine the person for signs of ischaemia and palpate the foot pulses [23]. In addition, age (year), Body Mass Index (kg/m^2^), Fasting Blood Glucose (mmol/L) and duration of diabetes (months) were collected as the patient related continuous measures.

#### 2.2.2 Foot and Wound‐Related Measures

Neuropathy was assessed [24, 25] while presentation delay, wound size and SINBAD classification [26] were recorded. The presentation delay was calculated as the number of days the foot ulcer existed (patient noticed the ulcer) before they presented to the service (the first appointment when treatment started).

Diagnosis of infection was conducted in accordance with the latest IWGDF practical guidelines on the prevention and management of diabetes‐related foot disease [23]. This was done by the presence of at least two clinical signs or symptoms of inflammation (redness, warmth, induration, pain/tenderness) or purulent secretions [23]. For clinically infected wounds, a tissue specimen was obtained by curettage or deep tissue biopsy. In case facilities of culture and sensitivity are not available, as might be the case in LMICs (which was the case in our study), Gram‐stained smear was conducted [23]. On the other hand, using a swab was avoided as recommended by the IWGDF practical guidelines [23].

### 2.3 Follow‐Up

The patients were followed up for 167 ± 408 days during which all patients received standard treatment. There were 99 out of the initial 1174 patients lost to follow‐up and 1074 patients stayed in the trial.

The diabetic foot nurse emphasised the importance of maintaining their regular foot care routines during checkups. Additionally, strict management of diabetes and high blood pressure was enforced through the concurrent use of oral hypoglycaemics and antihypertensive medications. Anti‐platelet medications were administered when necessary. Moreover, participants benefited from quarterly educational sessions conducted by the diabetic foot care nurse. Consistent glycaemic control was monitored by obtaining fasting or random blood glucose readings at each follow‐up appointment. Also advice on reducing the weightbearing activities was given consistently to all participants. The standard management of risk factors—such as tobacco use, diabetes duration, LDL levels and hypertension—was applied to all patients. Medical therapy and revascularization were provided when indicated, including the use of antiplatelet agents and ACE inhibitors. Additional risk‐factor control, including smoking cessation, lipid‐lowering therapy and blood‐pressure management, was implemented as required. Glycaemic control was monitored at each follow‐up visit using fasting or random blood glucose measurements.

In addition, for the pressure offloading and ulcer protection, a felted foam in combination with appropriate footwear for patients was considered according to the recommendation in the IWGDF practical guidelines [23]. This was compliant with the guidelines that recommend use of such, in the absence of TCC/on‐removable knee‐high offloading or other form of biomechanical relief device [23], that was the case in our study that was conducted in a low resource setting.

Wound debridement was provided according to the IWGDF 2023 guidelines on wound healing [27].

This included only sharp debridement as the standard of care as a minor chairside procedure performed in a clinical setting by a nurse to cut away necrotic tissue that is not viable. Autolytic, biosurgical, hydrosurgical, chemical, ultrasonic or laser debridement has not been used. The frequency of sharp debridement was determined by the clinician based on the clinical need of each patient, with small ulcers generally deemed as not requiring sharp debridement. All procedures were in accordance with the recommended IWGDF Guidelines on interventions to enhance healing of foot ulcers in people with diabetes [27]. Debridement was conducted by a trained nurse and identified at the same appointment when the wound dressing was changed where regular inspection of the ulcer was conducted according to the IWGDF practical guidelines on DFU care [23].

### 2.4 Data Analyses

Cox proportional hazard model was utilised to develop a prognostic model to predict ulcer healing and to identify variables that predict the risk of ulcer not getting healed. Kaplan Meier survival analysis was used to assess the effect of different variables on healing time. All analyses were performed using IBM SPSSv.29.0.1.0.

## 3 Results

From the total of 1075 (M/F: 664/441) patients with diabetic foot ulcer that were followed‐up for 167 ± 408 days, 1039 patients have had their ulcers healed and 36 remained unhealed.

Table 1 shows the categorical measures for the two groups of ulcers (healed ulcers vs. those that remained unhealed).

**TABLE 1:** The categorical measures of the two groups of healed and unhealed ulcers.

	Categorical variable	All (1075)		Healed ulcers (1039)		Ulcers not healed (36)	
		Count	%	Count	%	Count	%
Patient‐related measures	Female	411	38.2	394	37.9	17	47.2
	Previous ulceration history	663	61.7	637	61.3	26	72.2
	Amputation history	7	0.7	7	0.7	0	0
	Peripheral arterial disease	242	22.5	229	22.0	13	36.1
	Never smoked	846	78.7	818	78.7	28	77.8
	Past smoker	70	6.5	67	6.4	3	8.3
	Current smoke	159	14.8	154	14.8	5	13.9
	Neuropathy	1069	99.4	1033	99.4	36	100
Wound related characteristics	Amputation during treatment	15	1.4	12	1.2	3	8.3
	Ulcer area > 1 cm^2^	1020	94.9	985	94.8	35	97.2
	Deep ulcer	1068	99.4	1033	99.4	35	97.2
	Forefoot ulcer	660	61.6	643	62.1	17	48.6
	Wound debridement	965	89.8	932	89.7	33	91.7
	SINBAD 3	41	3.8	40	3.8	1	2.8
	SINBAD 4	497	46.2	486	46.8	11	30.6
	SINBAD 5	439	40.8	422	40.6	17	47.2
	SINBAD 6	98	9.1	91	8.8	7	19.4
	Right foot ulcer	564	52.5	547	52.6	17	47.2

Table 2 shows the continuous measures for the two groups of ulcers (healed ulcers vs. those that remained unhealed).

**TABLE 2:** The differences in continuous measures between the healed and unhealed group.

Total ulcers		Patient‐related measures						Wound related characteristics				
		Age (years)	Height (m)	Weight (kg)	BMI (kg/m^2^)	Diabetes duration (month)	FBG (mmol/L)	Wound length (mm)	Wound width (mm)	Wound size (mm^2^)	Delay in presentation (days)	Treatment duration (days)
Healed	Mean	57.2	1.64	75.0	27.9	116.5	14.4	41.2	30.1	2234.2	15.2	167.7
	Stdev	12.3	0.08	17.5	6.5	95.1	16.2	42.1	29.9	4931.5	37.6	412.0
Not Healed	Mean	56.6	1.61	70.3	27.2	109.8	14.0	71.4	53.6	6396.4	23.0	144.9
	Stdev	13.7	0.12	18.8	5.7	89.5	7.8	62.2	53.9	11508.5	62.5	270.2
Total	Mean	57.2	1.64	74.9	27.9	116.3	14.4	42.2	30.9	2370.1	15.4	166.9
	Stdev	12.3	0.08	17.6	6.5	94.9	16.0	43.2	31.2	5317.6	38.7	408.1

Cox proportional hazard analysis indicated that a regression model could predict non‐ulcer healing (*χ* ^2^ = 172.6; df = 11; *p* = 0.000). The developed hazard model showed to be able to predict the ulcer healing efficacy with the prediction power (AUC = 0.701[0.597–0.805]; *p* = 0.000) with 98.5% precision (positive predictive value), 68.1% sensitivity (recall), 67.6% specificity and an F1 score of 0.81 indicating a high performance.

**FIGURE 1:**
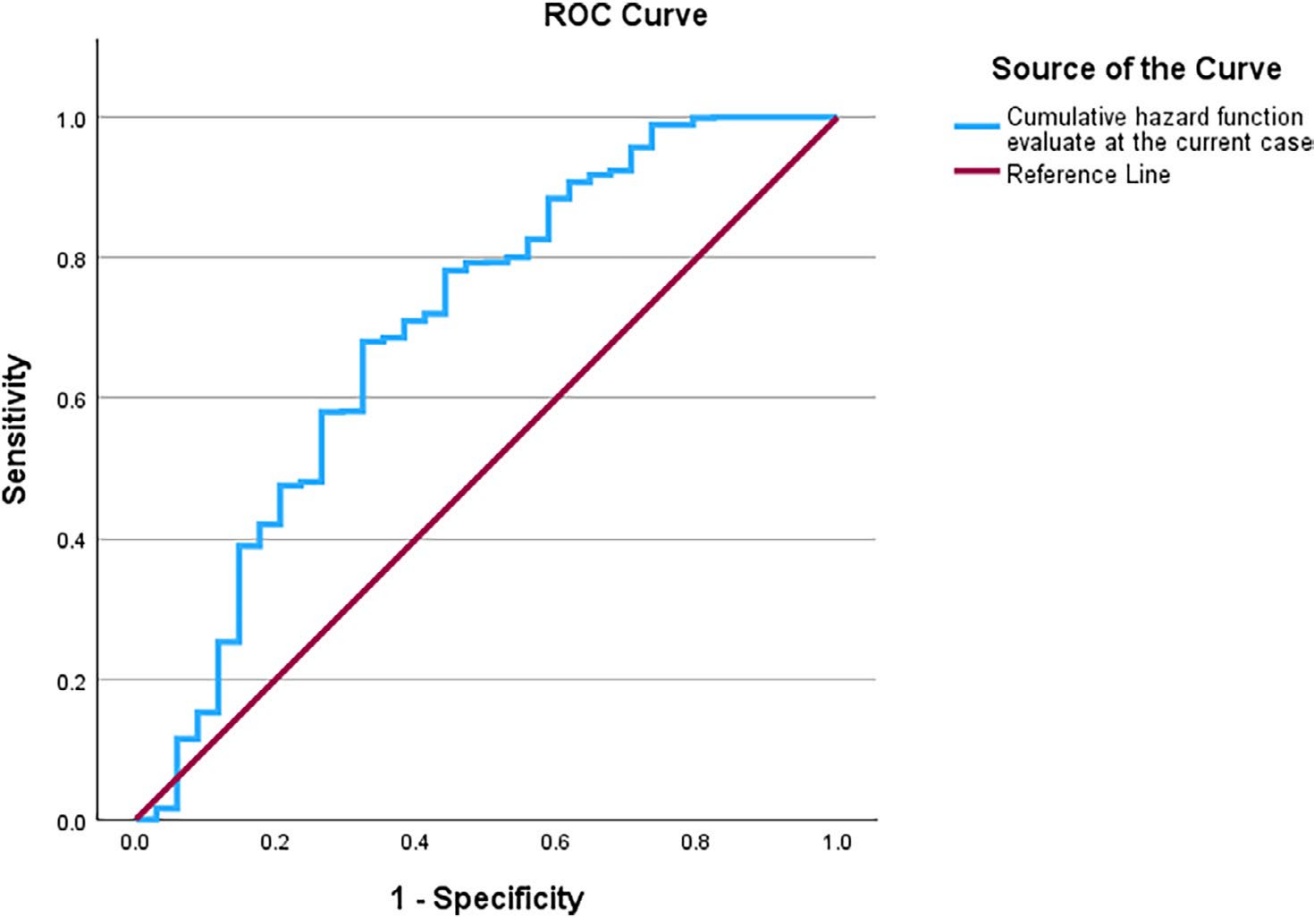
Receiver Operating Curve (ROC) for the Cox Proportionate Hazard Model.

Figure 1 shows the received operating curve (ROC) along with the Cox Proportionate Hazard Model.

### 3.1 Prognostic Markers for the Healing Outcome

The Cox Proportional Hazard model identified several categorical variables (factors) (HR [95% CI]) that were significantly associated with increased risk of DFU not healing (Table 3).

**TABLE 3:** Variables in Cox survival model—with the event as wound healing.

	*B*	SE	Wald	df	Sig.(*p* < 0.05)	Exp(*B*)	95.0% CI for Exp(*B*)	
							Lower	Upper
*Variables in the equation*								
FBG (mmol/L)	0.000	0.002	0.000	1	0.990	1.000	0.995	1.005
Wound size (cm^2^)	0.000	0.000	36.269	1	0.000	0.995	0.994	0.997
Age (years)	0.005	0.003	2.262	1	0.133	1.005	0.999	1.011
Duration of diabetes (month)	0.000	0.000	0.010	1	0.921	1.000	0.999	1.001
Delay in presentation (days)	−0.005	0.001	27.133	1	0.000	0.995	0.993	0.997
Sex (Female)	−0.019	0.074	0.065	1	0.799	0.981	0.848	1.135
PAD	−0.372	0.206	3.268	1	0.071	0.689	0.460	1.032
Smoking (reference currently smoke)			8.728	2	0.013			
Never smoked	−0.023	0.098	0.054	1	0.816	0.977	0.807	1.184
Previously smoked	−0.437	0.164	7.096	1	0.008	0.646	0.468	0.891
Previous history of ulcer	−0.634	0.286	4.933	1	0.026	0.530	0.303	0.928
Amputation history	−0.157	0.412	0.145	1	0.703	0.855	0.382	1.915
Amputation during treatment	0.498	0.317	2.474	1	0.116	1.646	0.885	3.062
Ulcer depth	−1.150	0.418	7.588	1	0.006	0.317	0.140	0.718
SINBAD‐3 (reference)			32.385	3	0.000			
SINBAD‐4	−0.662	0.174	14.526	1	0.000	0.516	0.367	0.725
SINBAD‐5	−0.911	0.178	26.132	1	0.000	0.402	0.284	0.570
SINBAD‐6	−1.159	0.226	26.275	1	0.000	0.314	0.201	0.489
Side	−0.047	0.064	0.543	1	0.461	0.954	0.843	1.081
Wound debridement	−0.219	0.106	4.286	1	0.038	0.803	0.988	0.653

*Note:* Negative *B* values indicate association with wound not healing.

The Cox Proportional Hazard model identified the following variables (HR [95% CI]): a greater wound size (0.995 [0.994–0.997]) and delay attending the foot clinic after DFU (0.995 [0.993–0.997]) were both significantly associated with DFU not healing.

The model identified the following variables (HR [95% CI]) that were significantly associated with reduction in DFU healing: A greater wound size (0.995 [0.994–0.997]); delay attending the foot clinic after DFU (0.995 [0.993–0.997]); history of smoking (0.646 [0.468–0.891]); ulcer depth level (deep vs. superficial) (0.317 [0.140–0.718]); history of previous DFU (0.530 [0.303–0.928]); and performing wound debridement during treatment (0.803 [0.653–0.988]); SINBAD score 4 (0.516 [0.367–0.715]); SINBAD score 5 (0.402 [0.284–0.570]); SINBAD score 6 (0.314 [0.201–0.489]), each relative to SINBAD 3; were all significantly (*p* < 0.05) associated with decreased DFU healing.

While it appears that wound debridement increases the chance of ulcer not healing (HR: 0.803), as presented in Table 1, the proportion of patients with bigger than 1 cm^2^ was much lower among ulcers that healed (94.8%), compared to the 97.2% in the ulcers that did not heal. In another word, debridement performed for 89.7% of patients whose ulcer healed, but for 91.7% of patients whose ulcer did not heal, mainly because the ulcers that healed were inherently smaller and did not require debridement (Table 1).

### 3.2 Prognostic Markers for Healing Time

**FIGURE 2:**
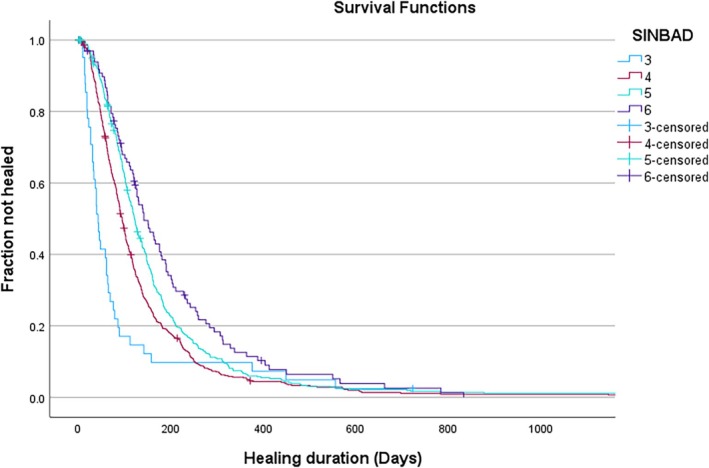
The fraction of ulcers not healed over healing time across SINBAD classifications.

Kaplan Meier survival analysis indicated that significant differences across SINBAD classifications (*χ* ^2^ = 38.431; df = 1; *p* = 0.000) in time to healing in days (Median [95% CI]) with SINBAD‐3 (43.0 [33.6–52.4]); SINBAD‐4 (95.0 [88.2–101.8]); SINBAD‐5 (122.0 [112.7–131.3]); SINBAD‐6 (142 [111.1–172.9]) as is shown in Figure 2.

**FIGURE 3:**
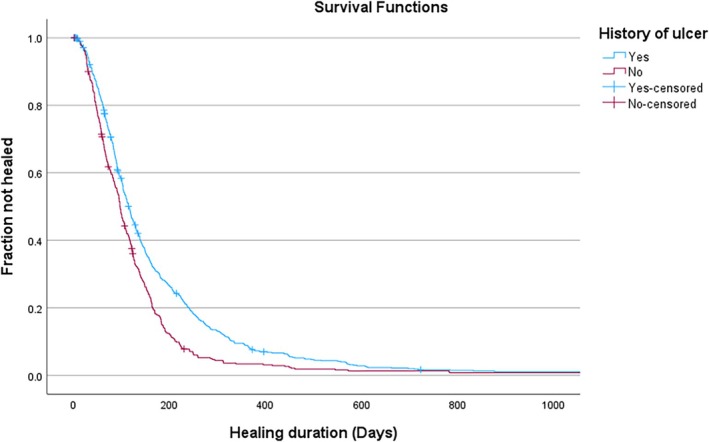
The fraction of ulcers not healed over healing time across with and with no previous ulcer history.

The time to heal was significantly different (*χ* ^2^ = 28.102; df = 1; *p* = 0.000) between those with previous history of ulcer (115.0 [106.7–123.22]) vs. those with no previous history of ulcer (96.0 [88.5–103.5]) as is shown in Figure 3.

**FIGURE 4:**
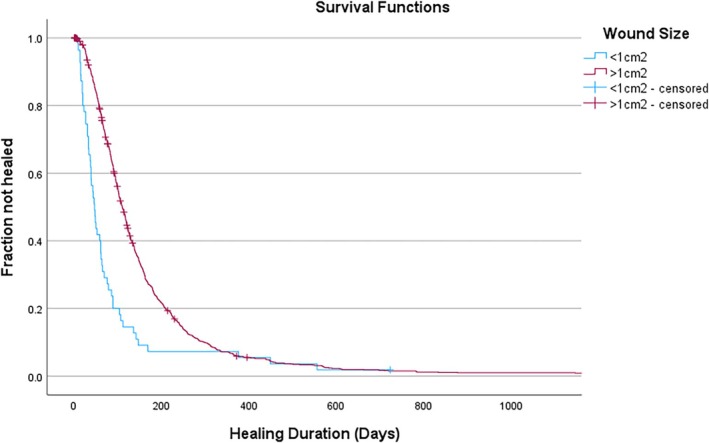
The fraction of ulcers not healed over healing time across wound size categories.

In addition, patients with deeper ulcers showed significantly (*χ* ^2^ = 9.343; df = 1; *p* = 0.002) longer time to heal (107.0 [100.9–113.1]) vs. those with superficial ulcers (68.0 [5.6–130.4]). Patients with bigger than 1 cm^2^ ulcers showed significantly (*χ* ^2^ = 26.826; df = 1; *p* = 0.001) longer time to heal (107.0 [101.0–116.0]) vs. those with smaller than 1 cm^2^ ulcers (47.0 [36.6–57.4]) as is demonstrated in Figure 4.

Patients with debridement had significantly (*χ* ^2^ = 9.233; df = 1; *p* = 0.002) longer time to heal (110 [103.8–116.2]) vs. those with no debridement (83.0 [68.6–97.4]). As highlighted above under 3.1 these results are related to the fact that bigger ulcers were associated with those that needed debridement and hence, because bigger ulcers take longer to heal, those that required debridement were also associated with longer healing time.

**FIGURE 5:**
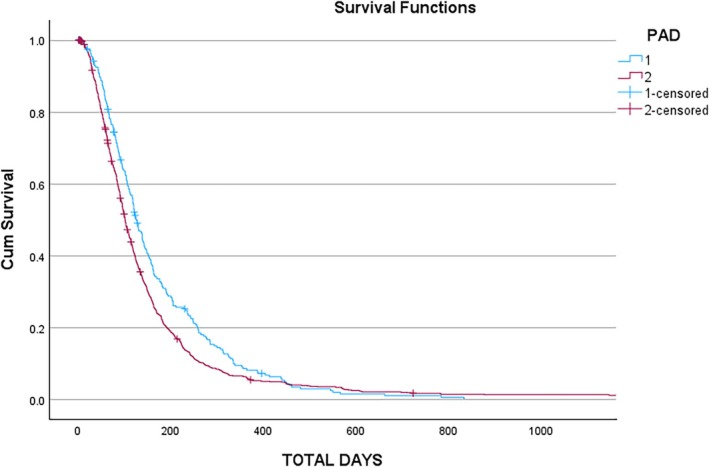
The fraction of ulcers not healed over healing time across PAD.

While peripheral arterial disease (PAD) showed a non‐significant (HR: 0.689 (0.460–1.032); *p* = 0.071), decrease in ulcer healing based on the Cox model, the Kaplan Meier survival analysis indicated a significant (*χ* ^2^ = 8.594; df = 1; *p* = 0.003) increase in time to heal in days (Median[95% CI]) for patients with PAD (125 [113.1–136.9]) compared with those with no PAD (101.0 [95.0–107.0]) as is shown in Figure 5.

### 3.3 Combined Effect of Categorical Variables (Factors) on Healing Time

When the analyses were formed on separate groups of patients with and with no history of previous ulcer, the following were observed. The higher SINBAD classification (*χ* ^2^ = 20.556; df = 3; *p* = 0.000 vs. *χ* ^2^ = 43.413; df = 3, *p* = 0.000) and a wound size bigger than 1 cm^2^ (*χ* ^2^ = 13.902; df = 1; *p* = 0.000 vs. *χ* ^2^ = 18.483; df = 1, *p* = 0.000) each led to a significant decrease in wound healing efficacy across both groups including with and without previous history of ulcer respectively. However, having wound debridement (*χ* ^2^ = 9.371; df = 1; *p* = 0.002) or PAD (*χ* ^2^ = 5.007; df = 1; *p* = 0.025) significantly increased the wound healing only in the group with previous history of ulcer, and not in the group without a history of previous ulcer (*χ* ^2^ = 1.67; df = 1; *p* = 0.196) and (*χ* ^2^ = 0.787; df = 1; *p* = 0.375) respectively. In contrast, ulcer depth significantly increased the wound healing (*χ* ^2^ = 11.625; df = 1; *p* = 0.001) only in patients with no previous history of ulcer and not in the group with previous history of ulcer (*χ* ^2^ = 2.453; df = 1; *p* = 0.117).

When the effect of PAD in patients with a history of ulceration was assessed, Kaplan Meier survival analysis indicated that a significant (*χ* ^2^ = 5.007; df = 1; *p* = 0.025) increase in time to healing in days (Median [95% CI]) for patients with PAD (139 [117.7–160.3]) compared with those with no PAD (106.0 [96.6–115.4]). However, there was no significant effect of PAD on healing duration in patients without a previous history of ulcers.

## 4 Discussion

### 4.1 Prognostic Markers for DFU Healing Outcome

The developed hazard model showed to be able to predict the ulcer healing efficacy with a clinically acceptable level of prediction power (AUC = 0.70) with 98.5% precision (positive predictive value), 68.1% sensitivity (recall), 67.6% specificity and with an overall accuracy of 68% with a F1 score of 0.81. This indicates that in general the ulcer healing outcome of 68% (more than 2 out of 3) patients can be accurately predicted. The high precision of 98.5% indicated that only 11 patients that were predicted by the model to heal did not heal (False Positive). On the other hand, a recall (Sensitivity) of 68% indicated that 332 patients who were predicted as not healing by the model healed (False Negative). While in the case of ulcer healing a False Negative could be considered as less costly compared to a False Positive, it can be concluded that the model is worthwhile overall. This is dictated by the fact that the model could correctly predict the ulcers outcome of 707 (out of 1039) patients who healed and 25 (out of 39) patients who remained unhealed.

The results of the current study in which wound classification was found to be a significant predictor of DFU healing outcome is generally in line with previous studies [8, 9].

However, in the current study an increase in wound classification showed to have the highest increase in the risk of ulcer not healing. Compared to SINBAD 3, the chance of wound not healing increased from 50% in SINBAD‐4, to 60% in SINBAD‐5 and to 70% in SINBAD‐6. This is an important finding as for the first time the wound classification is quantitatively associated with the risk of ulcer not healing. This can be important to assess the wound in relation to healing outcome and has practical implications in triage of diabetic foot ulcers in resource restricted settings.

This is particularly important and indicates that the SINBAD classifications can be used to evaluate the chance of non‐healing and to predict the outcome of diabetic foot ulcer healing. This complements the recommendations by the IWGDF wound classification guideline, where SINBAD as a simple, validated system was recommended for communication and audit [13].

In addition, this goes further and provides further support for the notion that the SINBAD system can also predict outcomes for an individual ulcer when used in conjunction with other measures such as delay in presentation and history of previous ulcer, as highlighted in Table 3. This is significant as, for the first time, there are clear indications that the SINBAD score can also be used to compare the chance of non‐healing i.e., by 50% when comparing SINBAD‐4 against the SINBAD‐3 classification. This is specifically important with clear practical implications, as the findings show that a SINBAD 3 wound would have twice the chance of getting healed compared to a SINBAD‐4; hence, triage could be tuned towards optimising the allocation of resources to each classification in a stratified fashion.

In addition, particularly ulcer depth was the strongest predictor of risk of ulcer not healing (68% increase in risk), which is in line with the results of Eurodiale study [7] where ulcer depth was a significant predictor of ulcer healing outcome, but contrary to a previous study in UK population where the depth of the ulcer and healing outcome were not significantly associated [28].

Ulcer depth characterised as deep versus shallow wound appears to have a very strong effect, similar to the comparison between SINBAD‐6 vs. SINBAD‐3, showing a 70% increase in risk of ulcer not healing.

A bigger wound size was found to significantly (*p* < 0.05) increase the risk of diabetic foot ulcer not healing, that is in line with previous studies [8, 9, 14]. The results of this study, where debridement showed to be associated with increased risk of ulcer not healing, may appear to be against the IWGDF guidelines that promote the use of sharp debridement as standard of care to promote ulcer healing [27]. However as indicated in the previous sections, it needs to be considered that in this study ulcers that needed debridement were inherently bigger, hence because of the strong association between ulcer healing and wound size, debridement also shows association with ulcer healing.

While our finding on the effect of wound size on healing is also generally in line with the results of the Eurodiale [7] our model predicts that by increasing the ulcer size by 1 cm^2^ the chance of ulcer not healing increases by 0.5%.

We also found that each 1 day delay in presentation (attending the clinic after ulcer occurrence) reduces the chance of ulcer healing by 0.5%, that is generally in line with previous studies indicating that the delay in presentation was associated with ulcer healing outcome [14]. Specifically, a delay of between 4 and 8 weeks was associated with a 25% increase in chance of ulcer not healing [14] that is broadly in line with our findings in the current study. Similarly, in a study on a small cohort of patients with DFU, the duration of diabetic foot ulcer (DFU) before seeking care (delay in presentation) was reported as predictors of healing outcome [29] that is in line with the findings of the current study. In the current study, PAD was not found to be associated with ulcer healing outcome, that is in line with another study [8], but contrary to the other previous studies [7, 9], in which PAD was reported to be associated with healing outcome.

It needs to be emphasised that for example Eurodiale study [7] only considered the outcome of ulcer healing at certain time point (12 months) as a measure whether an ulcer healed or not. Hence the results of our current study in which a survival analysis over time is used could not be directly compared against previous studies where a certain time‐point was used to assess healing.

It also needs to be emphasised that our finding indicated that Peripheral arterial disease (PAD) on its own cannot be used as a prognostic marker to predict the healing outcome of DFU, which is in line with the findings of the systematic review of literature in this area [30]. However, we did find that PAD significantly influences the healing time which is further discussed in the next section.

### 4.2 Prognostic Markers for DFU Healing Time

In relation to time to heal, wound classification showed the highest effect. More specifically in reference to SINBAD‐3, the healing duration significantly increased by 2.2, 2.8 and 3.3 folds in SINBAD‐4, SINBAD‐5 and SINBAD‐6 ulcers. In general this is in line with a study of a small patient population where the University of Texas DFU classification was associated with the ulcer healing time [29].

The IWGDF guidelines recommend SINBAD as a reliable ulcer classification system for population‐level auditing, due to its practicality and consistency across settings [13]. The findings of the current study for the first time extend the application of SINBAD beyond the audit and communication and in comparing the ulcer healing time in a cohort of patients. For example, knowing that it takes a SINBAD‐6 ulcer more than 3 times (3.3) to heal compared to SINBAD‐3 would allow optimisation of resource allocation and a quantitative evidence‐based approach to assessing the adequate time of treatment.

This again brings the IWGDF guideline recommendations to a next level where for the first time the wound classification is linked to ulcer healing in a prospective setting.

Specifically, it took more than 2.4 times longer for ulcers bigger than 1 cm^2^ to heal compared to those smaller than 1 cm^2^. This shows that ulcer area was the strongest predictor of healing duration after SINBAD scores and it appears that its effect (2.4 times healing duration for bigger ulcers) is even stonger in comaprionto SINBAD‐4 (vs. SINBAD‐3) that increases the duration of healing by 2.2 times.

Afterwards, having deeper ulcers (compared to superficial), wound debridement, Peripheral Arterial Disease and history of previous ulcer increased the time to healing by 57%, 32%, 24% and 20% respectively. Very few studies investigated the association between different clinical measures and healing time of DFU. Majority of studies considered the healing outcome over a fixed study period, that is, 12 months [7] or did not report the healing duration even when the healing outcome over variable time duration was studied [14].

With subgrouping the patients into those with and without a previous history of ulcer, the SINBAD classification and wound size showed to have affected the ulcer healing duration across both groups with and without previous history of ulcer. However, having PAD and wound debridement seem to have increased the healing duration only in patients with a previous history of ulcer and ulcer depth seems to have only influenced the healing duration in patients without a previous history of ulcer. These variations could have practical implications in stratifyig patients for resource allocation.

It needs to be highlighted that as described under 2.5, the shorter duration of healing for a small portion of ulcers that did not go through debridement was due to the fact that those ulcers were inherently smaller and did not need debridement to be performed.

### 4.3 Strength and Limitations

The present study is unique as it reports on a cohort of patients who were followed for a longer period of 167 ± 408 days to identify the prognostic markers for ulcer healing efficiency. This is one of the few studies in which a diverse number of measures as potential prognostic markers were included, which allowed the influence of variables on the risk of wound not healing and on the time to heal to be identified.

The model showed to have a high precision of 98.5%, indicating that only 11 patients that were predicted by the model to heal did not heal (False Positive). On the other hand, recall (Sensitivity) was only 68%, indicating that 332 patients who were predicted as not healing by the model eventually healed (False Negative). While in the case of ulcer healing a False Negative could be considered as less costly compared to a False Positive, it needs to be taken into account that in implementation for larger population other factors such as the overall cost consequence to the healthcare system need to be considered [31].

Despite the inclusion of commonly collected clinical data, adding tissue wound perfusion or more specific measures of ankle brachial pressure index using hand‐held doppler could be useful in future to assess the healing efficiency of DFU. All assessments were conducted in accordance with the IWGDF practical guidelines on the prevention and management of diabetes‐related foot disease [23]. Considerations with regards to cost and the time it takes to conduct the measurements need to be considered specifically in low‐resource settings such as LMIC where access to specialised equipment such as hand‐held doppler may be limited. On the other hand, in future studies to look at improvements in foot screening are warranted as previous work has shown the effectiveness of such to reduce the burden of diabetic foot disease like amputation rates in a LMIC setting [32].

### 4.4 Clinical Implications and Future Directions for Healthcare Guidelines and Policies

Compared with a SINBAD score of 3, SINBAD scores of 4, 5 and 6 increased the risk of ulcer not healing by approximately 50%, 60% and 70%, respectively, and prolonged the time to healing by 2.2‐fold, 2.8‐fold and 3.3‐fold, respectively.

Particularly ulcer depth was the strongest predictor of the risk of ulcer not healing (68% increase in risk), while ulcer area was the strongest predictor of healing duration, which increased the duration of healing by 2.4 times.

This is clinically important for optimising resource allocation as the wound classification information could be used not only to establish the risk of not healing, but to assess the time it takes for the ulcer with different classifications to heal.

This can also be used for more effective treatment of an ulcer that doesn't heal or takes longer to heal in light of the wound classifications [10, 11, 12].

These findings highlight a clear, practical implication indicating that SINBAD can support a stratified approach in triage, helping clinicians prioritise resources and intervention intensity for patients at higher risk of poor healing. While SINBAD could not be regarded as a stand‐alone predictor of individual healing, it represents the most pragmatic and internationally applicable DFU classification system, offering the strongest balance between clinical relevance and feasibility [13]. This could have implications in further advancing the IWGDF recommendations [27] by linking wound classification directly to prospective healing outcomes, providing clinicians with a simple, evidence‐based tool to stratify care intensity and optimise clinical workflow. This is also in line with recent study in which SINBAD has demonstrated clinically meaningful associations with major adverse foot events, supporting its utility for standardised risk stratification [33].

Deeper ulcer and ulcer history showed to increase the hazard of ulcer not healing by 68% and 47% respectively and increased the healing duration by 57% and 20%.

Specifically, it took more than 2.4 times longer for ulcers bigger than 1 cm^2^ to heal compared to those smaller than 1 cm^2^. Afterwards, having wound debridement, Peripheral Arterial Disease increased the time to healing by 32% and 24%, respectively.

This finding of the study has significant clinical implications specifically in limited resource settings such as Low Middle‐Income Countries [34]. Diabetes imposes a heavy burden on the health services in most African countries; the findings of this study have major implications in developing policies for low resource settings. For example, we found that for each day delay in presentation (attending the clinic after ulcer occurrence) there is a 0.5% reduction in chance of ulcer healing. The same reduction rate in ulcer healing happens for an increase in the ulcer size by 1 cm^2^.

Considering these findings, the DFUs can be stratified based on their healing efficiency, where limited resources can be best allocated to the wounds with a higher chance of remaining unhealed, considering the effects on healing duration and outcome. The outcome of this work has direct implications for health care practices, reimbursement policies and gross domestic products in low resource settings.

### 4.5 Concluding Remarks

This prospective study indicated that the SINBAD wound classification was the most influential factor, being associated with both a reduced likelihood of healing (by at least half −50%) and a prolonged time to heal (‐by at least more than twice 2.2 times) as long for a unit increase in SINBAD score. Ulcer with SINBAD‐6 score was associated with a 70% reduced likelihood of healing and a 3.3 times increase in time to heal compared to ulcers with SINBAD‐3 score. These highlight important indicators which allow comparison of prognosis of ulcers at different severities.

Among SINBAD constitutive components, ulcer depth (shallow‐vs‐deep) was the strongest predictor of risk of ulcer not healing, while ulcer area (bigger or smaller than 1 cm^2^) was the strongest predictor of time to heal. In addition to wound classification, delay in presentation was associated with the decreased healing chance which needs to be considered in predicting ulcer outcome.

## Funding

The authors have nothing to report.

## Ethics Statement

The study received ethical approval from the School of Health and Society Research Ethics Committee, University of Salford (Ref: 5159). This study used secondary anonymised data collected at Abbas Medical Centre, Dar es Salaam, Tanzania and all participants gave informed consent before taking part.

## Conflicts of Interest

The authors declare no conflicts of interest.

## Data Availability

The data that support the findings of this study are available on request from the corresponding author. The data are not publicly available due to privacy or ethical restrictions.
